# Self-assembled xK1 rosette nanotube as a nanocarrier for chemotherapeutic drug molecules: a molecular dynamics study

**DOI:** 10.1039/d5ra04657b

**Published:** 2025-11-28

**Authors:** Hanah Nasifa M. Ali, Arthur A. Gonzales

**Affiliations:** a De La Salle University 2401 Taft Avenue 0922 Manila Philippines; b Chemical Engineering Department, University of the Philippines – Diliman Quezon City 1101 Philippines aagonzales1@up.edu.ph

## Abstract

Chemotherapy is widely recognized as an effective treatment for various types of cancers, relying on chemotherapeutic drugs such as chlorambucil (CBL), camptothecin (CPT), doxorubicin (DOX), and flutamide (FLU) to inhibit cancer cell metastasis through their cytotoxic effects. However, these drugs also interact with noncancerous cells, causing significant side effects, including but not limited to nausea, vomiting, hair loss, peripheral neuropathy, lymphedema, and infertility. Developing nano-based drug vehicles is one approach to alleviate such adverse effects. This study introduces a three-ring-xK1-type rosette nanotube (RNT) as a potential nano-based drug-delivery vehicle for these chemotherapeutic agents. Molecular dynamics (MD) simulations aided by the molecular mechanics/three dimensional reference interaction site model (MM/3DRISM) analysis were employed to evaluate the potential of xK1 as a drug carrier. Results revealed that xK1 exhibited structural stability and strong binding affinity with the selected drugs under physiological conditions, with binding energies ranging from −4.66 to −26.10 kcal mol^−1^. The drugs favorably bind at either end of xK1 due to the prominent aromatic rings facilitating π–π stacking interactions. Furthermore, MD simulations of the release mechanism revealed structural disassembly, validating the responsiveness of the systems under acidic conditions. The findings here offer insights into the viability of xK1 for targeted drug delivery, which warrants further experimental studies.

## Abstract 2

Self-assembled xK1 exhibited stable binding with chemotherapeutic drugs (CBL, CPT, DOX, and FLU) under physiological conditions and demonstrated structural disassembly at low pH levels, suggesting its potential as a targeted drug delivery system.

## Graphical abstract

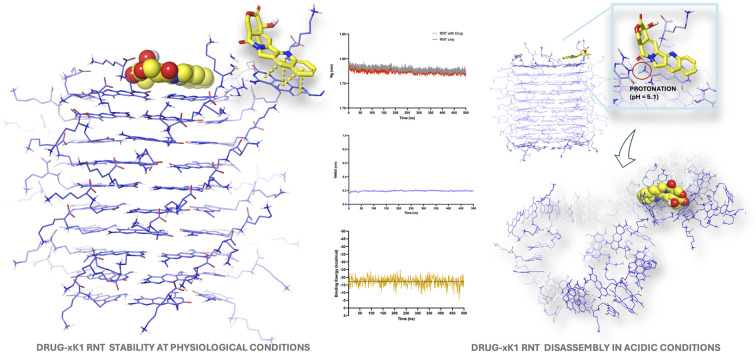

## Introduction

The main cancer treatment modality utilized worldwide is chemotherapy, where drugs are intravenously or orally administered to cause cancer cell death. The primary goal of chemotherapy is to use the cytotoxic effects of drugs to halt the spread of cancer cells. However, it also affects non-cancerous cells, causing adverse side effects on patients. Reports indicate that this treatment significantly reduces the quality of life for cancer patients, with physical symptoms—particularly chemotherapeutic side effects—being the strongest factor.^1^

Efforts have been made to alleviate the severe side effects of chemotherapeutic drugs by employing strategies, such as combining them with bacterial metabolites,^2^ regulating redox balance using natural products,^3^ utilizing natural medicines to treat side effects,^4^ and implementing cryotherapy to mitigate the side effects during chemotherapy.^5^ One approach is to develop nano-based drug vehicles, which can alleviate the severe side effects by enabling controlled drug release, protecting the healthy cells from exposure, crossing the biological barriers to target cancer cells directly, and improving the stability and biocompatibility within the bloodstream.^6–8^ Numerous studies have investigated the application of nanotechnology in drug vehicles, including carbon-based nanotubes or nanosheets,^9–11^ liposome-based^12^ and chitosan-based^13^ nanomaterials, carrier proteins,^14^ gold nanoparticles,^15^ metal-based nanoparticles,^16^ quantum nanodots,^17,18^ and others.^7,19^ A study reported the suitability of chitosan nanoparticles as drug vehicles owing to their physicochemical stability and bioavailability.^20^ Dendrimers have also gained increasing attention as drug-delivery vehicles owing to their physicochemical features, such as low polydispersity, nano-scale dimensions, high solubility, and the ability to conjugate various molecules through their branched cavities and surface groups.^21^ A significant challenge in the delivery of chemotherapeutic drugs is their limited water solubility. This is commonly addressed by modifying the hydrophobic carriers through hydrophilic attachments or surface functionalization. Among nano-based drug vehicles, carbon-based drug vehicles have been extensively studied. However, these systems can cause kidney injuries during the excretion process, particularly with larger-sized molecules.^22^ Additionally, studies have shown that they tend to accumulate in organs, such as the lungs, liver, spleen, and kidneys, for prolonged periods, potentially leading to long-term toxicity.^22,23^ Thus, there is a need for a nanocarrier that is biocompatible and has a high therapeutic efficacy.

**Fig. 1:**
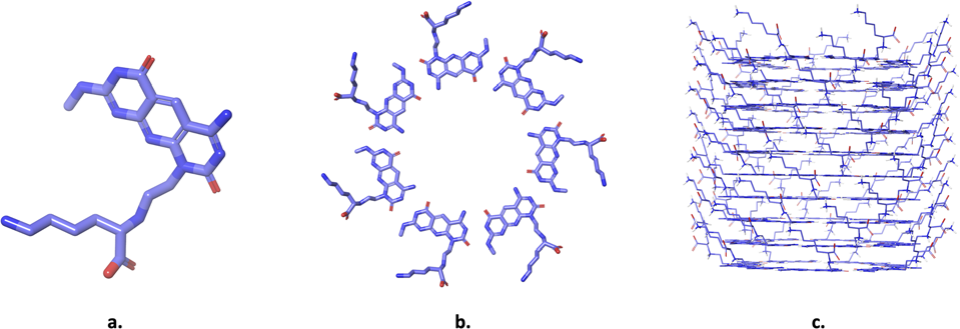
Molecular model of rosette nanotube displaying the (a) motif molecule of xK1, (b) top view of the nanotube, and (c) front view of the nanotube.

This study explores the potential of rosette nanotubes (RNTs) as a drug vehicle. RNTs exhibit significant properties, such as biocompatibility for biomedical applications, amphiphilicity, low toxicity, and self-assembly behavior, making them potential candidates for the delivery of chemotherapeutic drugs.^24^ These properties are useful for enhancing the loading, delivery, and solubility of hydrophobic drugs in aqueous environments. RNTs are supramolecular structures composed of self-assembling motifs designed from guanine (G) and cytosine (C) base pairs. These base pairs display complementary hydrogen bonding patterns, namely donor–donor–acceptor for guanine and acceptor–acceptor–donor for cytosine. Through self-assembly, the G∧C motif (Fig. 1a) forms rosette-shaped rings (Fig. 1b), which subsequently stack into stable, hollow nanotubular structures (Fig. 1c) *via* forces such as hydrophobic interactions, dispersion forces, and stacking interactions.^25,26^ A key feature of RNTs is their modifiable surface, which can accommodate a variety of functional groups. This feature enhances their solubility and bioavailability.^24^ The tubular design of RNTs includes a hydrophobic inner core that can encapsulate hydrophobic drugs, while the functionalized outer surface protects the drugs in physiological environments, enabling controlled drug release.^27,28^ In addition, the terminal regions of the RNTs feature prominent aromatic rings, which can interact with drugs containing aromatic moieties. This characteristic further enhances the potential of the system as an efficient drug-delivery vehicle. In this study, the xK1 type of rosette nanotube (RNT) is utilized. The xK1 (Fig. 1c) type of RNT was designed through supramolecular engineering of its G∧C-based motif formed by the same G and C hydrogen bonding arrays as the bicyclic ring, but placed with a pyridine ring in between.^25^ This was designed to achieve control over the structural parameters of the rosette nanotube (RNT). The incorporation of the pyridine ring allows the fine modulation of π–π stacking and hydrogen-bonding interactions between the rosette subunits, resulting in an increased inner diameter and enhanced hydrophobicity of the internal cavity. This tunability in both geometry and surface chemistry provides a crucial advantage for the accommodation of drug molecules of varying sizes and polarity, making xK1 a more versatile and efficient drug-carrier RNT platform. The defining characteristic of xK1 lies in its enhanced hydrophobicity and amphiphilic tubular structure, which strongly facilitates the formation of assemblies in water.^25^

The limited biomedical applications of drug nanocarriers are primarily attributed to their poor biocompatibility, renal toxicity, and long-term accumulation in the body, as discussed. In contrast, rosette nanotubes (RNTs), which self-assemble through hydrogen bonding of G∧C motifs, offer a safe and biocompatible alternative. Experimental studies successfully reported the applications of RNTs as drug vehicles of hydrophobic dexamethasone for bone regrowth,^28^ tamoxifen as an anti-cancer drug,^29^ and siRNA for gene silencing.^30^ RNTs have also been utilized for tissue engineering applications as a scaffold for neural regeneration, nerve repair^31^ and cartilage repair.^32^ These studies suggest that RNTs are highly suitable for biomedical applications, specifically as a drug vehicle for hydrophobic anti-cancer drug molecules.

This study aimed to investigate the structural and energetic stability of xK1 under physiological conditions as a drug vehicle for chemotherapeutic agents, such as chlorambucil (CBL), camptothecin (CPT), doxorubicin (DOX), and flutamide (FLU). Most chemotherapeutic drugs are hydrophobic in nature and known to be cytotoxic. Molecular dynamics (MD) simulations aided by robust energetic analysis were employed in this study to evaluate the potential of xK1 as a targeted drug delivery system for chemotherapeutic molecules. This approach has been employed in investigating the stability and viability of a system consisting of drug-nanocarriers, as demonstrated in a recent study on zwitterion/PAMAM/CQD hybrid nanocarrier for doxorubicin (DOX).^33^ The authors investigated the interactions and stability of the hybrid nanocarrier under physiological conditions by employing MD simulations and energetic analysis. Another recent study by Alkriz and Joujeh^34^ evaluated the potential of RNA aptamers as drug vehicles for chemotherapeutic drugs by employing computational experiments, including molecular dynamics simulations. Thus, the approach in this study is suitable for investigating xK1 as a drug vehicle for chemotherapeutic drugs. Additionally, this approach offers a more efficient and cost-effective alternative to *in situ* experiments, providing molecular-level insights that are difficult to achieve experimentally. MD simulations allow the comprehensive analysis of stability and molecular interactions between drug molecules and RNTs, which are crucial for understanding the behavior of the drug vehicles under specific conditions. Additionally, energetic analysis using the Molecular Mechanics Poisson Boltzmann Surface Area (MM/PBSA) model and the Three-Dimensional Reference Interaction Site (3DRISM) model provides the system's energies, offering significant support for the overall analysis. MD simulations were also performed under acidic conditions to gain insights into the release mechanisms of the complexes. The results here collectively offer valuable insights into the viability of xK1 as a targeted drug vehicle for chemotherapeutic agents. The findings from this study are essential for developing safe and effective drug-delivery formulations. To the best of our knowledge, this is the first study to investigate xK1 as a drug vehicle for chemotherapeutic drugs, such as CBL, CPT, DOX, and FLU.

## Experimental

### System preparation

The xK1 type of RNT used in this study was modelled and prepared using Schrödinger Maestro 2021-1.^35^ The motif molecule was optimized using the Generalized AMBER Force Field (GAFF) and subsequently multiplied into a rosette ring structure, which was further stacked in a tubular configuration. The modelling and parameterization of the RNT structure were adapted from established studies^24,36^ and are detailed in SI Fig. S1.

The molecular structures of the drug molecules (CBL, CPT, DOX, and FLU) were retrieved from PubChem^37^ and ChemSpider.^38^ The system topologies were prepared using the GAFF parameters with partial atomic charges assigned *via* the AM1-BCC method generated using ACPYPE.^39^ This allows the use of GAFF parameters in GROMACS while maintaining compatibility with AMBER-based tools, such as gmx_MMPBSA, thereby ensuring methodological consistency throughout all simulation and analysis stages. GAFF parameters were also employed for various nanocarriers^40–43^ in various applications. In addition, each motif molecule was derived from guanine and cytosine bases, which were developed into a new monomer structure. This resulted in another molecule that required parameterization as a novel small molecule. GAFF, an extension of the AMBER force fields designed for drug-like and organic molecules, was therefore employed.^44^ GAFF covers a broad chemical space, including common atom types, such as C, N, H, F, and Cl. Since CBL and FLU consist of Cl and F atoms, respectively, GAFF parameters are particularly suitable for them.

### Molecular docking

#### Binding regions

**Fig. 2:**
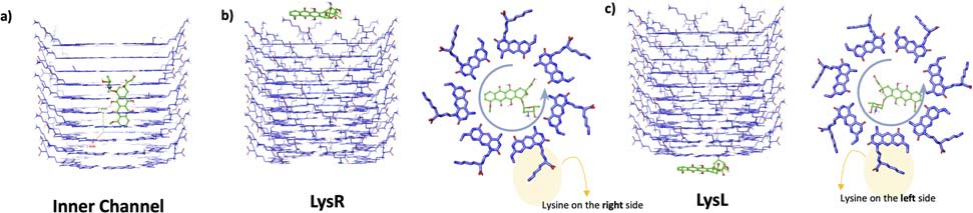
Accessible binding regions of xK1, where the drug molecules are (a) in the inner channel, (b) on the LysR end, and (c) on the LysL end.

Three accessible binding regions are specified for each drug: (1) in the inner channel, (2) at one end with lysine on the right side, and (3) at one end with lysine on the left side. The first configuration (Fig. 2a) is when the drug molecule is in the inner channel. The orientation is described based on the alignment of the longer side of the drug molecule along the *x*- and *y*-axes. If the longer side of the drug is aligned along the *x*-axis in the inner channel of xK1, then it is deemed horizontally positioned. If the longer side is aligned along the *y*-axis in the inner channel of xK1, then it is deemed vertically positioned. The orientation of each drug molecule along the inner channel of xK1 is based on the results from the molecular docking studies.

The two remaining binding regions are based on the ends of the channel of xK1. These regions are labeled according to the position of lysine (Lys) when viewed from the top in a counterclockwise direction. In one configuration (Fig. 2b), Lys appears on the right side of each motif within the xK1 and is labeled as LysR. In the other configuration (Fig. 2c), Lys appears on the left side of each motif within the RNT and is labeled as LysL.

#### Docking parameters

Autodock4.2 tool^45^ was used for molecular docking, both for global and local docking. The systems were prepared using AutoDockTools-1.5.7 (ADT) prior to docking. The drugs were treated as flexible systems, while the xK1-RNT was kept rigid. The grid box dimensions and coordinates were adjusted to ensure that all accessible surfaces of the xK1 were enclosed. For local docking, a specific region was defined. Both global and local docking were utilized in this study.

The grid box for both global and local docking was centered at coordinates 50 × 50 × 49.609 Å with a grid spacing of 0.375 Å. A cubic grid box with grid points of 126 × 126 × 126 was used to define the binding sites for global docking. All drugs in the global docking revealed favorable poses at the LysR region (SI Fig. S2). Hence, local docking at LysL and within the inner channel was subsequently employed (SI Fig. S3 and S4). For the region at LysL, the grid box was defined with grid points of 110 × 78 × 26, while for the region within the inner channel, the grid box was defined with grid points of 68 × 48 × 70. The binding poses were analyzed based on the estimated binding free energy per pose. The top-ranked pose within the defined binding regions was selected as the initial state for each complex before MD simulation.

### Molecular dynamics

Molecular dynamics (MD) simulations were performed using GROMACS 2024.2 (ref. 46). Complex systems comprising xK1-RNT and one drug molecule (CBL, CPT, DOX, or FLU) were prepared through energy minimization, solvation, and ion addition, employing GAFF parameters. The energy minimization step utilized the steepest descent algorithm, while the solvation process employed the TIP3P water model. A NaCl ion concentration of 0.15 M was added to maintain physiological ionic conditions. Equilibration was carried out in two phases. In the first phase, an *NVT* ensemble (isothermal–isochoric or canonical) was used to stabilize the system's temperature. This was followed by an *NPT* ensemble to stabilize the system's pressure. A V-rescale thermostat with a time constant of 0.1 ps was employed for temperature control, while pressure regulation was achieved using the Berendsen barostat with a 1 ps time constant and a compressibility of 4.5 × 10^−5^ bar^−1^. After equilibration, a 500 ns production simulation with an integration time step of 2 fs was performed in the *NPT* ensemble. All bonds involving hydrogen atoms were constrained using the LINCS algorithm. A force-switch modifier was applied to the van der Waals interactions between 1.0 and 1.2 nm. Long-range electrostatic interactions were calculated with the Particle Mesh Ewald (PME) method. The temperature was maintained at 310 K (37 °C) using the velocity-rescaling thermostat (V-rescale) with a coupling constant of 0.1 ps. Pressure coupling was controlled by the Parrinello–Rahman barostat, with isotropic scaling at 1 bar, a time constant of 2.0 ps, and an isothermal compressibility of 4.5 × 10^−5^ bar^−1^. The temperature was set at 310 K to mimic physiological conditions. Each complex system comprised one selected chemotherapeutic drug (CBL, CPT, DOX, FLU) in three different configurations within xK1 obtained from molecular docking. This provided insights into the stability of each complex system under the designed physiological conditions (310 K, 0.15 M NaCl). Each system with the same initial states was executed in triplicate.

#### Protonated system

Cytosine-rich fragments are reported to protonate with the change in pH.^47–49^ They are reported to fold and unfold in acidic and neutral pH environments. Thus, with the change in pH, cytosine-rich fragments can trigger the release of drug molecules to the target cancer cells. G∧C motifs of xK1, having cytosine as a fundamental component, are hypothesized to be able to release drug molecules into the target cancer cells. The G∧C motif for this system was protonated to mimic an acidic environment. N_2_ depicted in SI Fig. S1 for each selected motif molecule was protonated after the MD simulation of a complex system under physiological conditions.

The fraction of protonated cytosines for each RNT was determined using the fraction protonated equation (eqn (1)). The reported p*K*_a_ of cytosine is approximately 4.5,^50^ and the tumor microenvironment typically exhibits a pH of 5.1. Based on the calculation, approximately 20% of the motifs within each RNT required protonation. Accordingly, 14 motifs of xK1 were protonated as structurally represented in SI Fig. S5. The protonated rings at the termini of xK1 were selected due to their accessibility within the environment. The structural response of each RNT under these acidic conditions was investigated.1
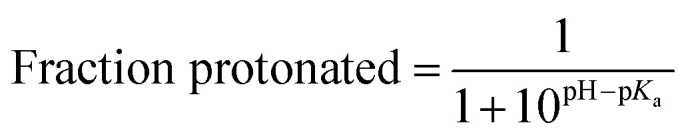


The simulation time for this system was set at 100 ns, and the same simulation parameters described above were consistently applied to ensure consistency across all systems.

### Post-MD analysis

In the post-simulation phase, the following analyses were performed: (1) radius of gyration (*R*_g_), (2) root mean square deviation (RMSD), (3) detailed interaction analysis, and (4) radial distribution function (RDF) analysis. *R*_g_ was employed to evaluate the compactness and stability of each complex system throughout the simulation. It measures how far the atoms of a molecule are spread out from the center of mass. In this study, the radius of gyration (*R*_g_) was analyzed for both xK1 alone and the xK1–drug complex. A peak in the *R*_g_ profile of the complex indicates the movement of the drug molecule away from xK1, suggesting structural instability or a transition of the drug from one binding region to another. RMSD analysis was also employed to evaluate the stability of each complex system throughout the simulation. This method evaluates structural changes from the initial configuration to the final state, with an equilibrating RMSD plot indicating structural stability. RDF analysis measures the radial distribution of the selected atoms at a given distance from a set of reference atoms. In this study, the selected atoms were from water molecules, while the reference atoms were taken from the motif molecules within the binding regions, including the drug molecules. RDF analysis was employed to evaluate the hydrophobicity of the complex systems. *R*_g_, RMSD, and RDF analyses were performed using GROMACS.

Detailed interaction analyses were conducted using Schrödinger's Maestro interaction analysis tool to identify additional interactions contributing to the binding of the drug molecule and xK1. This approach offers a comprehensive understanding of the interaction that stabilizes the complex system.

### Energetic analysis

#### MM/PBSA for stability analysis

Energetic analysis was performed to further evaluate the stability of the drug molecule's association with xK1 throughout the simulation period. The Molecular Mechanics Poisson–Boltzmann Surface Area (MM/PBSA) method was employed to estimate the effective binding free energy of each drug–xK1 complex after system stabilization, as determined from the radius of gyration (*R*_g_) analysis following molecular dynamics (MD) simulations. Binding free energies were calculated for every 10th frame of the trajectory to monitor fluctuations and assess the temporal stability of the interaction. In these calculations, xK1 was defined as the receptor and each drug molecule as the ligand.

MM/PBSA calculations were employed here using the gmx_MMPBSA tool. The tool integrates molecular dynamics (MD) trajectories generated with GROMACS into the AMBER-based MM/PBSA.py workflow.^51^ Specifically, AmberTools20 (ref. 52) was employed alongside GROMACS for this computational analysis. The workflow of the gmx_MMPBSA tool is typically divided into three key stages: (1) preparation, (2) calculation, and (3) analysis. During the preparation stage, GROMACS topologies are converted into AMBER-compatible topologies using ParmEd.^53^

The total free energy of each system can be expressed by eqn (2), where *E*_MM_ represents the molecular mechanics energy, *G*_solvation_ denotes the solvation free energy, and −*TS*_solute_ corresponds to the entropic contribution. The overall binding energy is calculated as the difference between the total energy of the complex and the sum of the energies of the isolated RNT and drug molecule, as shown in eqn (3). Accordingly, the total free energy consists of both enthalpic (Δ*E*_MM_ + Δ*G*_solvation_) and entropic energy (eqn (4)). The *G*_solvation_ is decomposed as polar and non-polar solvation energies, where the polar energy is estimated using the Poisson–Boltzmann model (eqn (5) and (6)).

2 *G* = *E*_MM_ + *G*_solvation_ − *TS*_solute_

3 Δ*G*_bind_ = *G*_complex_ − (*G*_RNT_ + *G*_drug_)

4 Δ*G*_bind_ = Δ*E*_MM_ + Δ*G*_solvation_ − *T*Δ*S*_solute_

5 Δ*G*_solvation_ = Δ*G*_polar_ + Δ*G*_nonpolar_

6 *G*_solvation_ = Δ*G*_PBSA_ + Δ*G*_nonpolar_

7 Δ*G*_bind_ = Δ*E*_MM_ + Δ*G*_solvation_

MM/PBSA calculations mainly account for the enthalpic energy without the entropic contribution (eqn (7)). Thus, binding affinities from MM/PBSA calculations are referred to as effective binding energies.^54^ The entropic term can be estimated through separate methods, such as normal-mode analysis; however, it was excluded in this study due to its high computational cost. Furthermore, the calculation of entropic contributions is computationally demanding, requiring extensive sampling and significant memory resources. Limited snapshot sampling often leads to large fluctuations in entropy estimates, and achieving statistically stable predictions would necessitate a prohibitively large number of frames.^55,56^ Since the primary objective of this work was to compare relative binding energies over time, the entropic contribution was not included in this stability analysis.

#### MM/3DRISM for binding energies

The binding energies of the drug molecules against xK1 were quantified using MM/3DRISM analysis. 3DRISM was combined with molecular mechanics calculations using the gmx_MMPBSA tool again. 3D-RISM is a statistical-mechanical solvation model that treats the solvent as a continuum distribution of molecular interaction sites. It inherently accounts for the entropic contributions of solvation, providing a detailed thermodynamic evaluation of binding energies.

For 3DRISM, water was treated as the solvent, while the complex system was considered the solute. Water's solvent site–site correlation functions were computed using RISM theory coupled with the Kovalenko–Hirata (KH) closure approximation.^57^ The solvent-site distribution functions were then obtained on a three-dimensional grid around the complex system by solving the 3DRISM/KH equations using the site–site correlation and potential functions between the solute and the solvent. These were computed on a uniform 3D rectangular grid of 256 × 256 × 256 (ref. 36) within a supercell of 128 × 128 × 128 Å, providing a sufficient buffer of ≈15 Å on each side to fully solvate the structures. These dimensions ensured an adequate solvation space for reliable energetic estimates. A single trajectory protocol (STP) approach was used, where the bound and unbound states of the receptor and ligand were derived from the same trajectory, thus generating zero-bonded energies (Δ*E*_bonded_ = 0). As mentioned above, the total binding energy (eqn (7)) is the sum of its molecular mechanical energy (Δ*E*_MM_) and solvation energy (Δ*G*_3DRISM_). Nonbonded energies, specifically van der Waals energy (Δ*E*_vdW_) and electrostatic energy (Δ*E*_elec_), mainly contribute to the Δ*E*_MM_ (eqn (8)). The free solvation energy is calculated from the 3DRISM model (eqn (9)).

8 Δ*E*_MM_ = Δ*E*_bonded_ + Δ*E*_nonbonded_ = Δ*E*_vdW_ + Δ*E*_elec_

9 Δ*G*_solvation_ = Δ*G*_3DRISM_ = Δ*ε*_solv_ − *T*Δ*S*_solv_

The 3DRISM model inherently accounts for both the solvation energy (Δ*ε*_solv_) and the solvent entropic contribution (−*T*Δ*S*_solv_). A negative solvent entropic contribution indicates positive entropy, implying a favorable and more disordered system thermodynamically. The overall binding energy comprises nonbonded energies and the energetic components from the 3DRISM calculations, as described in eqn (10).

10 Δ*G*_bind_ = Δ*E*_vdW_ + Δ*E*_elec_ + Δ*ε*_solv_ − *T*Δ*S*_solv_

## Results and discussion

### System stability under physiological conditions

Molecular dynamics (MD) simulations with structural and energetic analyses were performed to study the potential of the xK1 type of RNT as a drug vehicle for chemotherapeutic drugs. The molecular behavior, structural stability, and energetic stability were evaluated under physiological conditions to elucidate the potential of xK1 as the carrier. In addition, the structural changes of xK1 in an acidic environment were examined to provide insights into the release mechanism of the drugs from xK1. Global and local docking were employed to identify the initial states of each complex system subjected to MD simulations. Top-ranked binding poses revealed from global docking were utilized, which revealed that all drugs have a high binding affinity at the LysR (SI Fig. S2). Hence, local docking at the LysL and inner channel was performed subsequently. Accordingly, the top-ranked pose at the LysL and inner channel were utilized (SI Fig. S3 and S4), separately.

**Fig. 3:**
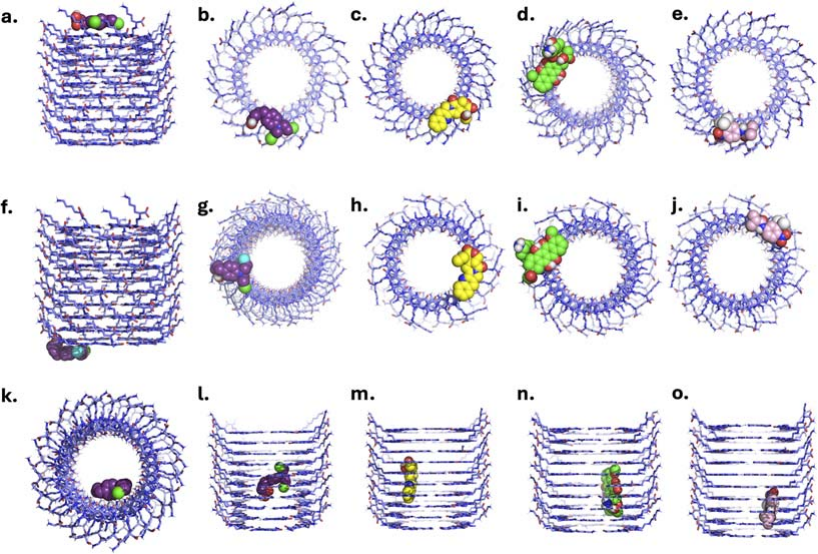
Initial configurations of each complex system obtained from molecular docking. (a) Front view of the CBL positioned at LysR. Top views of the same binding site for (b) CBL, (c) CPT, (d) DOX, and (e) FLU. (f) Front view of CBL positioned at LysL, with corresponding top views for (g) CBL, (h) CPT, (i) DOX, and (j) FLU. (k) Top view of CBL initially positioned within the inner channel. Front views of the same binding site for (l) CBL, (m) CPT, (n) DOX, and (o) FLU.

Shown in Fig. 3 are the top-ranked poses of each drug molecule within the specified binding regions (LysR, LysL, and inner channel). The drugs at both ends are observed to lie directly on the rings and not within the hollow channel. Each drug molecule exhibits diverse conformations at the ends of xK1 (Fig. 3a–j). On the other hand, each drug within the inner channel occupies a unique position and orientation. CBL (Fig. 3k and l) is horizontally inclined in the middle of the inner channel but closer to the rings than to the middle of the hollow channel. CPT (Fig. 3m) is positioned vertically in the center, closer to the rings than the hollow channel. DOX (Fig. 3n) is closer to the LysL end in a vertical position. Similarly, FLU (Fig. 3o) is closer to the LysL end, but it is slightly inclined vertically. These are the initial states of each complex system subjected to MD simulations under physiological conditions.

**Fig. 4:**
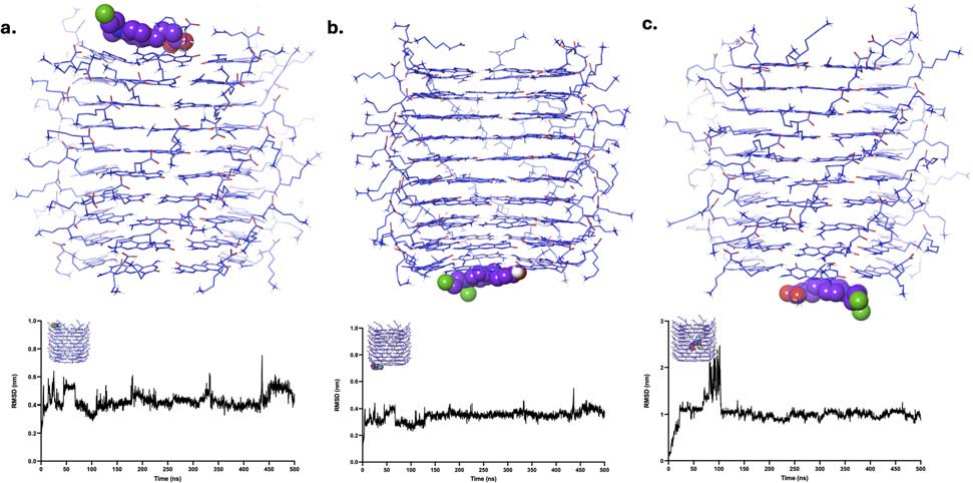
Clustered structures of the CBL–xK1 complex after MD simulations. The clustered MD trajectories correspond to systems where CBL was initially positioned at (a) LysR, (b) LysL, and (c) the inner channel. The structures were clustered in their last 100 ns to indicate the final states of each complex system. The plots below each structure show the corresponding RMSD values calculated specifically for the localized region where the drug molecule was initially placed.

Visual inspection revealed that CBL, initially at the LysR and LysL regions, remained equilibrated in its respective domains, as evidenced by Fig. 4a, b and SI Videos S1, S2. RMSD analysis (Fig. 4a and b) demonstrated small fluctuations (<0.3 nm), which are attributed to the diverse interactions of FLU at various motifs within the LysR and LysL regions. Despite these small fluctuations, the intactness and stability of CBL within the LysR and LysL, respectively, are preserved. In contrast, CBL transitioned and interacted at the LysL end of xK1, as evidenced in Fig. 4c and SI Video S3. The RMSD plot (Fig. 4c) exhibited a significant increase in peak (from 2 nm to 3 nm) around 100 ns during the transition. However, the equilibration of the plot occurred when CBL interacted at LysL. The equilibration of CBL at both ends of xK1 demonstrated a significant interaction between CBL and the termini compared to the inner channel.

**Fig. 5:**
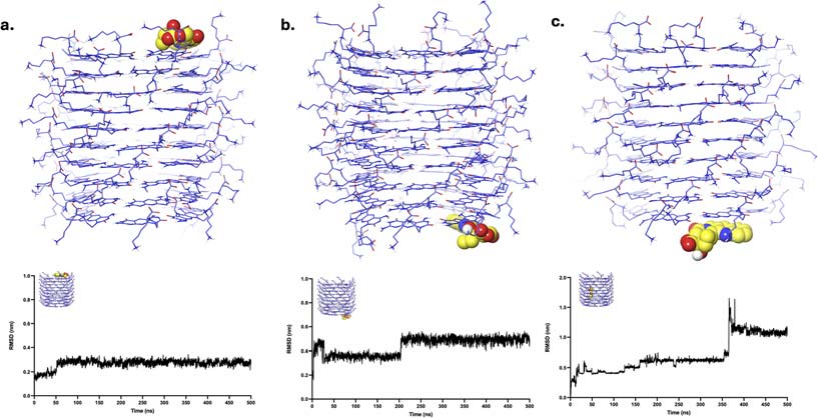
Clustered structures of the CPT–xK1 complex after MD simulations. The clustered MD trajectories correspond to systems where CPT was initially positioned at (a) LysR, (b) LysL, and (c) the inner channel. The plots below each structure show the corresponding RMSD values calculated specifically for the localized region where the drug molecule was initially placed.

CPT, when initially placed at LysR and LysL, remained in its position throughout the simulation, as evidenced by its MD trajectories (Fig. 5a, b and SI Videos S4, S5). RMSD analysis revealed the stability of the system throughout the simulation as it maintained its initial state configuration until the end of the simulation. Occasional fluctuations (<0.1 nm) are observed, primarily due to a brief transition of CPT from one motif to the next within the initial state. Similar to CBL, CPT transitioned and equilibrated at the LysL end of xK1, as depicted in Fig. 5c and SI Video S6. Nevertheless, the transition was gradual, as evidenced by its RMSD analysis, which indicated a sudden surge in peak (≈1 nm) at approximately 350 ns, and the onset of equilibration was observed afterwards. The equilibration of CPT at the termini of xK1 suggests a stronger interaction at the ends compared to the inner channel.

**Fig. 6:**
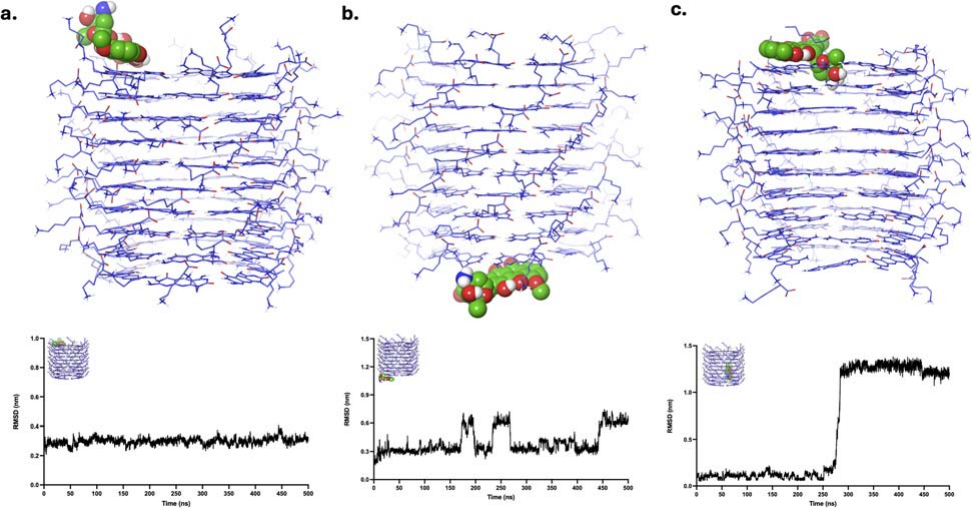
Clustered structures of the DOX–xK1 complex after MD simulations. The clustered MD trajectories correspond to systems where DOX was initially positioned at (a) LysR, (b) LysL, and (c) the inner channel. The plots below each structure show the corresponding RMSD values calculated specifically for the localized region where the drug molecule was initially placed.

DOX, when initially placed at LysR, remained in its position throughout the simulation, as evidenced by its MD trajectories (Fig. 6a and SI Videos S7). Similar to CPT, it maintained its initial state configuration throughout the simulation. The RMSD analysis revealed the plot's equilibration throughout the simulation, suggesting a stable binding of DOX to LysR while maintaining its configuration. DOX, initially bound to LysL (Fig. 6b and SI Video S8), remained in the binding site. However, it exhibited varying interactions across different motifs within the binding site, resulting in minimal fluctuations (<0.2 nm) in its RMSD plot. However, the minimal fluctuations suggest the intactness of DOX at the LysL. Analogous to CBL and CPT, DOX transitioned and equilibrated to one end of xK1 (Fig. 6c and SI Video S9). DOX equilibrated at the LysR end of xK1, resulting in an increased peak at 350 ns (≈1 nm), but an onset of equilibration was observed afterward. These findings suggest that DOX is favoured at the ends of the channel rather than in the hollow region of xK1.

**Fig. 7:**
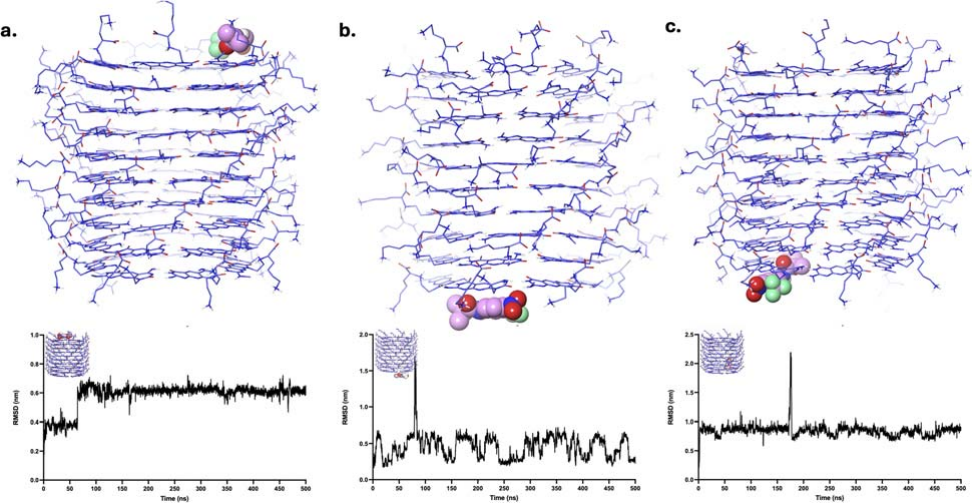
Clustered structures of the FLU–xK1 complex after MD simulations. The clustered MD trajectories correspond to systems where FLU was initially positioned at (a) LysR, (b) LysL, and (c) the inner channel. The plots below each structure show the corresponding RMSD values calculated specifically for the localized region where the drug molecule was initially placed.

The FLU drug molecule remained in the LysR end throughout the simulation (Fig. 7a and SI Video S10). Minor fluctuations are observed in the RMSD analysis, which are due to the shifts of the FLU from one motif to another motif of xK1 within the LysR end. FLU remained at LysL (Fig. 7b and SI Video S11), but it abruptly interacted with the bulk solution for a very short time around 90 ns, which resulted in a very abrupt spike in its RMSD analysis. Frequent small fluctuations (<0.3 nm) are also observed in its RMSD due to frequent shifts of FLU from one motif to another in the LysL region throughout the simulation. Meanwhile, FLU swiftly transitioned from the inner channel to the LysL of xK1, resulting in a sudden peak in the RMSD at approximately 175 ns (Fig. 7c and SI Video S12). Notably, frequent transitions of FLU between different motifs are also observed after this transition. These transitions suggest that FLU possesses multiple metastable states at the termini of xK1. However, the fact that they did not further distance themselves from xK1 indicates strong binding of FLU within the termini of xK1.

Following each MD simulation, comprehensive analyses were conducted, encompassing stability, structural, and energetic evaluations. The radius of gyration (*R*_g_) was utilized to quantify the stability of each complex system. It measures the overall compactness or intactness of the complex system over time.

**Fig. 8:**
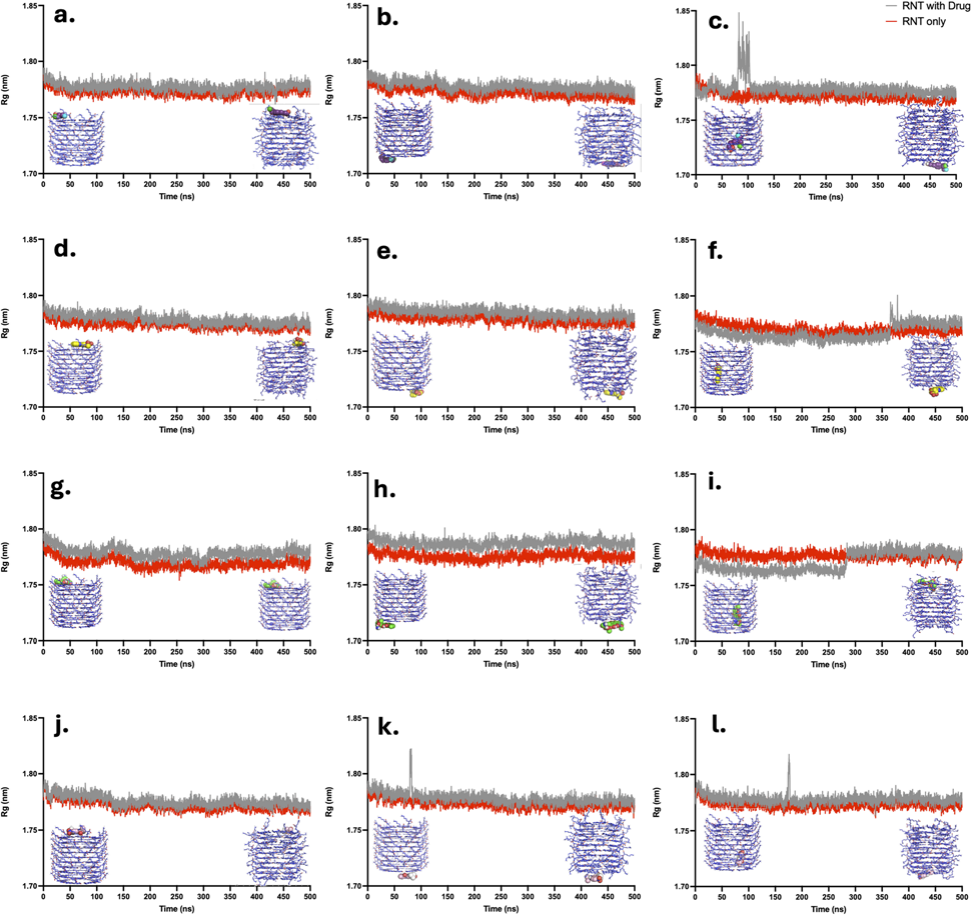
Radius of gyration of each complex system consisting of CBL (a–c), CPT (d–f), DOX (g–i), and FLU (j–l), initially at the LysR, LysL, and the inner channel of xK1, respectively. The initial states of each complex were obtained from the molecular docking study.

Additionally, root mean square deviation (RMSD) measurements were performed for the isolated drug, the isolated RNT, and the localized binding region, as previously discussed, to augment the structural analysis. The *R*_g_ plots (Fig. 8) reported herein show a comparison of the RNT alone (red line) and the complex system consisting of the drug and the RNT together (gray line). Thus, when the *R*_g_ for the complex system exhibits a sudden rise in its peak, it indicates that the drug has moved farther away from the RNT, suggesting the repulsion of the drug and, consequently, instability in the system.

Each drug molecule remained intact in its initial position at LysR throughout the 500 ns simulation (Fig. 8a, d, g and j). Similarly, CBL, CPT, and DOX remained stable at LysL throughout the simulation (Fig. 8a, e and h). FLU also exhibited overall intactness; however, the abrupt elevation observed in its plot (Fig. 8k) can be attributed to a brief interaction of FLU with the bulk solution around 100 ns. This phenomenon was transient, as FLU shortly re-associated with LysL. For systems where the drugs were initially positioned within the inner channel, the trajectories indicated a transition toward the terminal regions of xK1, resulting in a temporary rise in the corresponding *R*_g_ values. Despite this transient increase, the *R*_g_ later stabilized, indicating that the drugs achieved stable interactions at the terminal regions of xK1 following their initial confinement within the inner channel (Fig. 8c, f, i and l). Overall, the *R*_g_ analysis confirmed the stability of the drug molecules at the terminal regions (LysR and LysL). These findings correspond with the observed molecular behavior manifested in their MD trajectories.

The MD simulations were conducted in triplicate. The triplicate runs of the complex system comprising CPT and DOX demonstrated the consistency of their molecular behaviors, suggesting their stability from their initial states, particularly at the LysR and LysL ends, as observed in their *R*_g_ plots (SI Fig. S7 and S8). Similarly, these drugs, when initially positioned within the inner channel, consistently exhibited stabilization at the channel ends after transition, further indicating a strong interaction between CPT and DOX at the termini of xK1. This also suggests that once CPT and DOX attain their most stable configurations at the terminal regions of xK1, they are likely to remain bound there under physiological conditions.

In the case of CBL and FLU (SI Fig. S6 and S9), distinct molecular behaviors were observed across different simulation runs. In some trajectories, even when initially positioned at one end of xK1, both drugs eventually shifted toward the opposite end. Before shifting, each drug briefly interacted with the bulk solution but rapidly re-associated with the terminal regions, indicating a stronger affinity for the ends of xK1 compared to the surrounding solvent. This behavior suggests that CBL and FLU possess multiple metastable states at the terminal regions of xK1, leading to frequent transitions between motifs. Nevertheless, these observations still imply strong interactions, as no permanent dissociation or repulsion toward the bulk solvent was detected. Under realistic conditions, the xK1 structures exist as multiple connected fragments during drug delivery, so such movements could represent transitions of the drugs between carriers. The discussed MD trajectories in this paper were focused on the system that exhibited prolonged stability in their *R*_g_ and RMSD analyses. Moreover, RMSD analysis for each drug alone and RNT alone was conducted. The RMSD analyses consistently highlighted their structural stability throughout the simulations (SI Fig. S10 and S11). This indicates that RNT, as a carrier, is stable under physiological conditions.

**Fig. 9:**
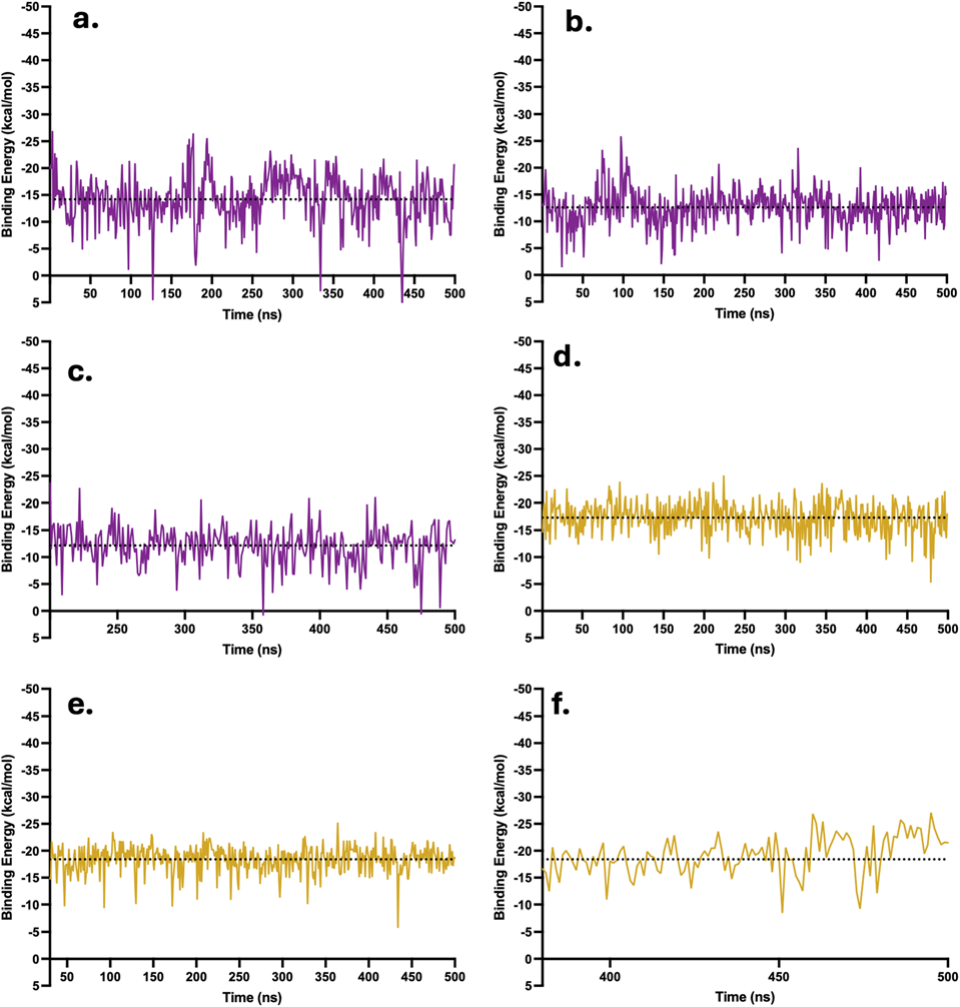
MM/PBSA binding energy plots of complexes in their equilibrated phase. The dotted line above the data points denotes the mean binding energy of each complex. Illustrated are the binding energies over time when CBL (a–c) and CPT (d–f) were initially at LysR, LysL, and the inner channel, respectively.

Plots of the binding energies by the MM/PBSA model were also obtained here for a robust stability analysis. The binding energies of each complex when they were stabilized were plotted over time. The binding energy profiles of CBL at LysR and after equilibration at LysL (from the inner channel) revealed occasional occurrences of positive binding energies. At LysR (Fig. 9a), transient spikes were observed at 128 ns (4.61 kcal mol^−1^), 334 ns (2.94 kcal mol^−1^), and 435 ns (1.27 kcal mol^−1^). Similarly, when CBL equilibrated at LysL, minor positive values appeared at 358 ns (0.65 kcal mol^−1^) and 475 ns (0.87 kcal mol^−1^). These brief positive fluctuations may correspond to the diverse interactions of CBL within the LysR and LysL regions, where the molecule transitions among motifs. Nevertheless, the predominance of negative binding energies throughout the trajectories indicates that the overall complexes remain stable, reflecting strong interactions across multiple configurations within the binding sites. This observation supports the earlier conclusion that CBL likely exists in multiple metastable binding states. In addition, (Fig. 9a, b and c) the distribution of energy in the plots for CBL was highly concentrated around the mean of each complex (−14.19, −12.6, and −12.16 kcal mol^−1^), respectively. This indicates that CBL has a favorable binding affinity with the LysR and LysL of xK1.

The binding of CPT to both LysR and LysL was found to be stable, as evidenced by the consistently negative binding energy values throughout the simulation after stabilization. In contrast to CBL, CPT exhibits smaller fluctuations, which aligns with the observed behavior of CPT wherein it remained localized at one to two motif molecules throughout the simulation (SI Videos S4 and S5). For the CPT at the LysR complex (Fig. 9d), the distribution of data points was largely concentrated around the mean of −17.33 kcal mol^−1^, suggesting a stable interaction between CPT and the LysR binding site. Similarly, as shown in Fig. 9e, CPT at LysL exhibited consistently negative binding energies throughout the simulation, indicating a stable interaction. Likewise, the energy distribution was highly concentrated around the mean of −18.40, further supporting the stability of the CPT at LysL. Meanwhile, Fig. 9f illustrates that CPT, after equilibrating at LysR around 380 ns following its transition from the inner channel, also maintained negative binding energies, signifying a strong binding affinity. Overall, the consistent negative binding energies in the plots indicate strong and stable binding of CPT at LysR and LysL.

**Fig. 10:**
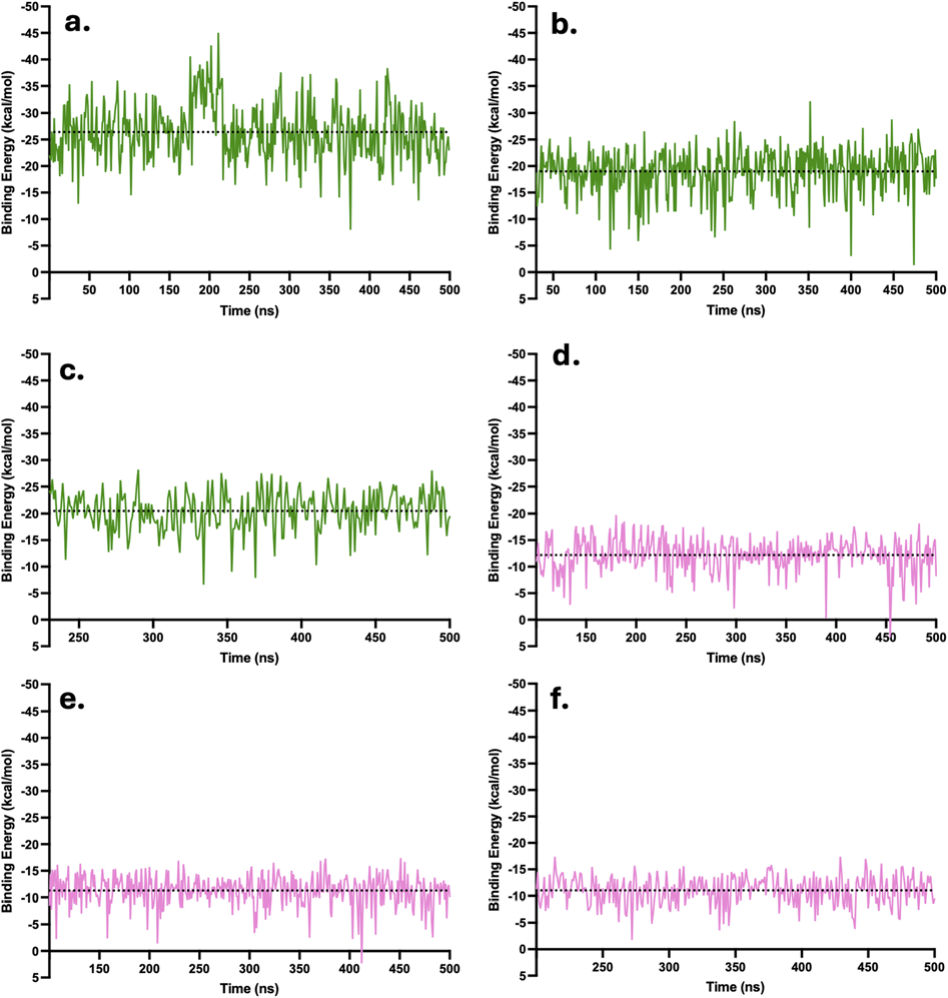
MM/PBSA binding energy plots of complexes in their equilibrated phase. The dotted line above the data points denotes the mean binding energy of each complex. Illustrated are the binding energies over time when DOX (a–c) and FLU (d–f) were initially at LysR, LysL, and the inner channel, respectively.

Among the drugs, DOX possesses the largest molecular size; consequently, its binding energy plots exhibited relatively broad intervals between fluctuations. Despite this, DOX consistently showed negative binding energies across all binding regions (Fig. 10a–c), indicating favorable and stable interactions at both LysR and LysL. Similar to CBL and CPT, the energy distributions were highly concentrated around their respective mean values (−26.41, −18.99, and −20.50 kcal mol^−1^), indicating the stability of the DOX at LysR and LysL of xK1. In contrast, FLU exhibited a few instances of positive binding energies (0.33 and 2.32 kcal mol^−1^), as shown in Fig. 10d and e. Nonetheless, the predominance of negative binding energies, like that for CBL, throughout the simulations suggests strong and stable associations of FLU at both LysR and LysL. The energy distributions were also concentrated around the mean values (−12.19, −11.28, and −11.03 kcal mol^−1^), further confirming complex stability. Overall, all drug molecules demonstrated strong and stable interactions at the LysR and LysL binding regions following equilibration. These findings highlight the suitability of xK1 as a potential drug delivery vehicle for CBL, CPT, DOX, and FLU, given its structural stability and sustained binding behavior under physiological conditions.

**Fig. 11:**
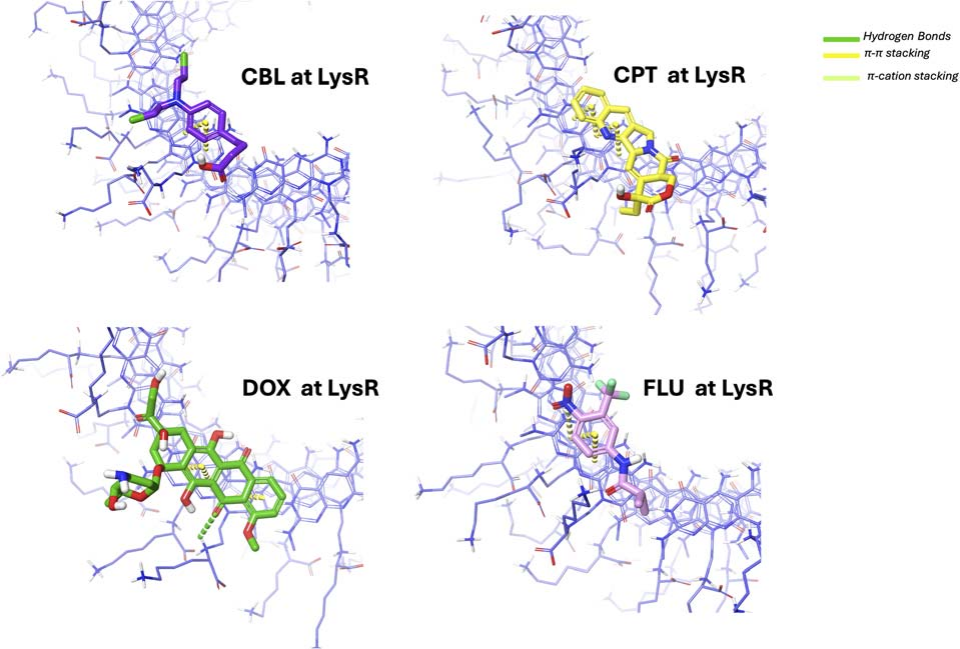
Structural interaction analysis of each drug with xK1 when they remained at LysR.

**Fig. 12:**
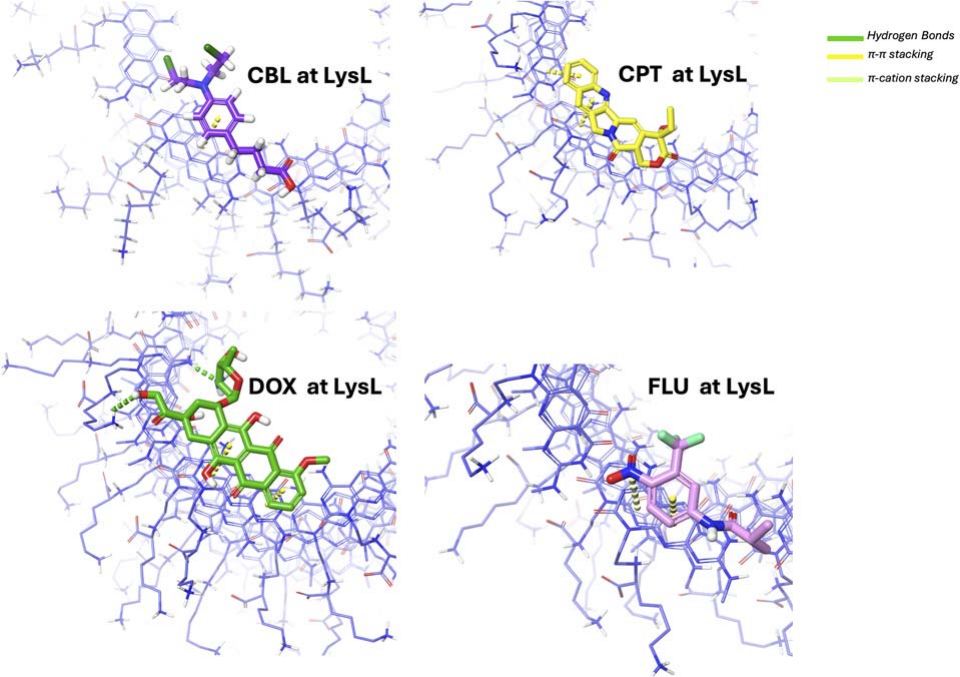
Structural interaction analysis of each drug with xK1 when they remained at LysL.

Further structural interaction analysis was conducted to identify additional interaction forces that contributed to the stability of the complex at the LysR (Fig. 11) and LysL (Fig. 12) regions. The π–π stacking orientations were observed for all complexes, as illustrated in Fig. 11. The π–π stacking interaction is anticipated due to the aromatic moieties of the drugs and the aromatic moieties at the end of xK1. They predominantly exhibited a parallel displaced arrangement. This orientation is known to contribute more substantially to system stability compared to other stacking configurations.^58^ DOX, aside from its π–π stacking interaction, forms a hydrogen bond with the terminal regions (LysR and LysL) of xK1. FLU was revealed to have a π–cation stacking of its nitrogen atom with the aromatic compound of xK1, both at the LysR and LysL regions. The π–cation interactions are recognized for their stronger stabilizing effect compared to π–π stacking interactions.^59^ Thus, the π–cation interaction played a critical role in enhancing the stability of the FLU–xK1 complex.

### Binding energies by MM/3DRISM

Energetic analysis using the Molecular Mechanics/Three-Dimensional Reference Interaction Site Model (MM/3DRISM) was employed to determine the binding affinity of the drug within the xK1 in their stable period. In the MM/3DRISM analysis for each system, the receptor is the RNT and the ligand is the drug molecule. The MM/3DRISM analysis was performed for the system in the equilibrated state. The equilibrated state based on the *R*_g_ analysis was clustered as one structure, and it was used as the reference structure for the analysis. The equilibrated state was based on the *R*_g_ analysis of each system. Systems with more negative binding energy values indicate stronger binding energy and a more stable complex.

It is observed that the solvation energy (Δ*ε*_solv_) for each complex is positive due to the nonpolar nature of both RNT and drug molecules. This indicates that each complex system has significant resistance to water. The main forces driving the interaction between xK1 and each drug are nonbonded and nonpolar solvation energies. Hydrophobic interactions play a crucial role, drawing the nonpolar drug molecule toward the nonpolar xK1 in the polar aqueous environment. Once the drug molecule is close enough, nonbonded interactions like van der Waals and electrostatic forces take over, as these are strongly dependent on the proximity of the interacting atoms. This is true and consistent for all complex systems. The entropic contribution (−*T*Δ*S*_solv_) represents the entropic forces arising from the solvent, primarily due to the displacement of water molecules upon the interaction of the drug with the binding site of xK1. A negative entropic energy (−*T*Δ*S*_solv_) corresponds to a positive entropy change, indicating a more thermodynamically favorable and disordered system. Therefore, a more negative entropic contribution reflects greater water displacement upon drug binding, signifying stronger binding affinity, enhanced stability, and a favorable contribution to the overall binding energy.

The MM/3DRISM analysis of all complex systems (Table 1) revealed strong binding energies of each drug molecule to the LysR and LysL sites. This aligns with the earlier observed molecular behavior and the obtained structural analysis. Most binding energies are more negative than the reported binding energy of a small drug molecule to xK1 (−5.81 kcal mol^−1^).^36^ When CBL and FLU transitioned from the inner channel to the LysL site, their resulting negative binding energies (−5.21 and −4.66 kcal mol^−1^) were weaker than those reported in a previous study. Nevertheless, since these energies are not significantly beyond the threshold, the negative values still indicate strong binding affinity. DOX consistently showed stronger binding energies (−24.10 kcal mol^−1^ and −16.25 kcal mol^−1^) at LysR and LysL than the rest of the drugs, which is likely due to its larger size than the rest of the molecules. The prominent aromatic rings at the ends of xK1 and each drug molecule, which facilitate π–π stacking interactions, favorably contribute to the van der Waals energy, which significantly contributes to the overall binding energy of each complex. Additionally, the entropic forces exhibited favorable contributions to the overall binding energy. The overall results suggest that these drugs have favorable binding energies with xK1, particularly at the ends.

**Table 1:** MM/3DRISM analysis of each complex system in kcal mol^−1^ units^a^

	Initial state	Final state	Δ*E*_vdW_	Δ*E*_elec_	Δ*ε*_solv_	−*T*Δ*S*_solv_	Δ*G*_bind_
CBL	LysR	LysR^b^	−26.61	−23.81	47.97	−6.48	−8.94
	LysL	LysL^b^	−26.52	12.87	9.22	−7.13	−11.56
	Inner	LysL	−16.93	−16.06	37.19	−9.40	−5.21
CPT	LysR	LysR	−32.77	7.18	25.14	−8.39	−8.85
	LysL	LysL	−30.03	−15.28	40.54	−7.96	−12.73
	Inner	LysR	−32.40	−8.33	41.32	−10.34	−9.74
DOX	LysR	LysR	−41.32	−19.28	48.51	−12.01	−24.10
	LysL	LysL	−38.50	−17.63	47.68	−7.80	−16.25
	Inner	LysL	−40.37	−17.35	57.67	−10.11	−10.16
FLU	LysR	LysR^c^	−21.48	−36.16	56.70	−6.20	−7.15
	LysL	LysL^b^	−21.54	6.74	14.00	−5.12	−5.92
	Inner	LysL^c^	−20.17	−23.61	41.57	−2.45	−4.66

a The selected system for the calculations is based on the stability observed from the *R*_g_ plots. The most stable plot in *R*_g_ was selected for further MM/3DRISM calculations, while the rest were calculated from their first simulation run.

b Calculations applied to the system from run 3 among the triplicate simulation runs.

c Calculations employed in the system from run 2 among the triplicate simulation runs.

As discussed, hydrophobic interactions significantly contribute to the binding of the drug molecules to xK1, due to their nonpolar nature. Radial distribution function (RDF) analysis was employed to quantify the hydrophobicity of the system, wherein the radial distribution of water molecules from the complex system was analyzed.

**Fig. 13:**
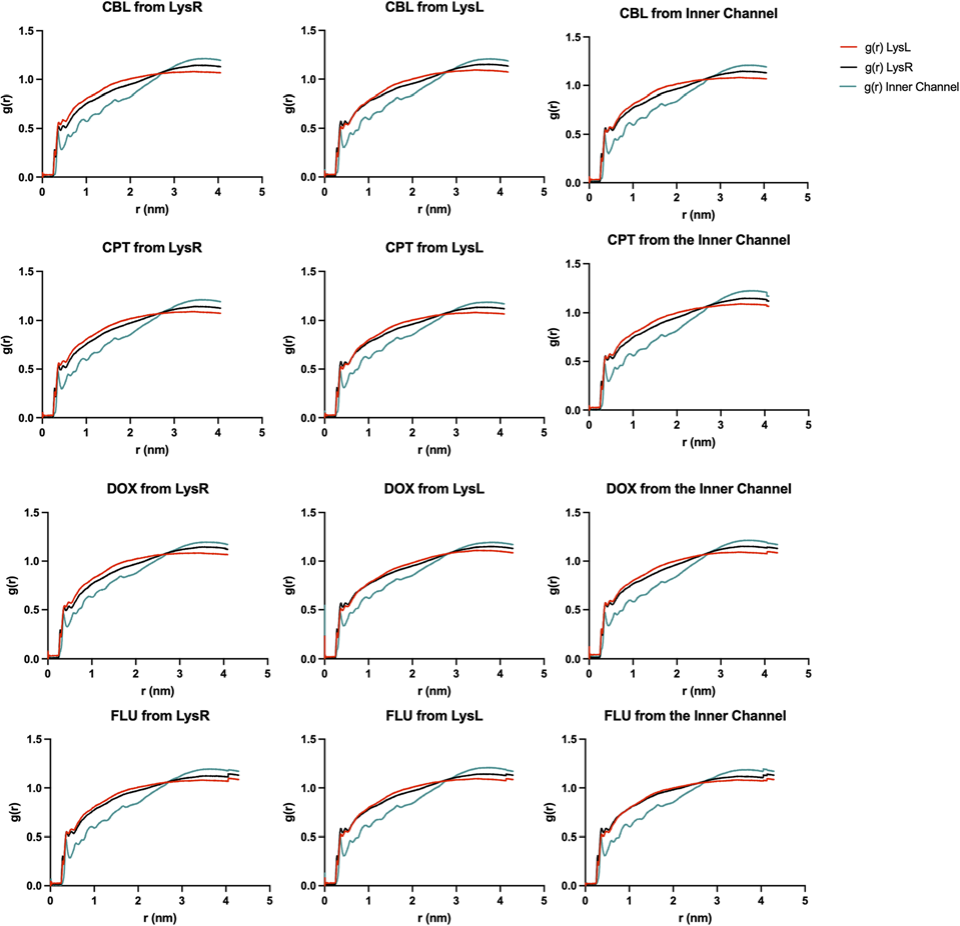
RDF analysis of water molecules at a distance from the complex system.

Fig. 13 illustrates the radial distribution of water molecules at a distance from the complex system. Each line in the plot represents a binding region of xK1, such as LysR, LysL, and the inner channel. In the analysis, the drug molecules together with the motifs from each binding region were selected as the reference atoms. The water molecules, on the other hand, were selected as the atoms to be evaluated. Fig. 13 demonstrates that water molecules consistently have a higher distribution at a farther distance of around 4 nm, indicating the repulsion of water from the complex. This quantifies and validates the hydrophobicity of each complex.

The reported structural and energetic analyses demonstrate that xK1 is a potential vehicle for chemotherapeutic drug molecules. All drugs, when initially at LysR and LysL, remain in their respective places. All systems also revealed that, starting from the inner channel, each drug molecule transitioned and eventually equilibrated at either end (LysR or LysL). This indicates that they are more successful on either end than in the inner channel of xK1. The equilibration of the drug molecules at LysR and LysL further supports findings from previous research, which suggest that xK1, in particular, lacks a significant potential energy barrier at either end.^36^ The potential energetic barrier is the energy that the molecule must overcome or pass through in order to be released from the inner channel of the RNT. This explains why the drugs were drawn by strong binding forces to the ends rather than remaining in the inner channel. In addition, most chemotherapeutic drugs contain aromatic rings, a fundamental component of RNTs that is more prominent at the ends. This characteristic contributes to their localization at either end due to π–π stacking interactions. Some papers reported that π–π stacking interactions increase the stability of the chemotherapeutic drugs with the carrier due to their aromatic compounds.^60–62^

It was discussed in a previous study that there are two potential loading mechanisms for the three types of RNT (K1, xK1, and iEt-xK1): (1) drugs will attach to either end after the stabilization of RNT, and (2) drugs will be encapsulated upon the self-assembly process.^36^ A similar loading mechanism is proposed in this paper, with the former mechanism being potentially more applicable for loading chemotherapeutic drug molecules in xK1-type RNTs. This is likely due to the prominent aromatic rings in xK1. As to the need to protect drug molecules from environmental exposure, particularly in noncancerous environments (*e.g.*, normal and healthy cells), the attachment of the drug vehicle at either end does not compromise its functionality in delivering the drug to the target cells. The strong binding energies and the observed structural stability of xK1 enhance its ability to shield the drug molecules from noncancerous cells, due to their strong associations (−4.66 kcal mol^−1^ to −24.10 kcal mol^−1^) with the vehicle. Several studies have reported the attachment of chemotherapeutic drugs to the surfaces of drug carriers, such as carbon nanotubes, graphene oxide, quantum dots, and other vehicles, which exhibit stability under physiological conditions.^9,62,63^ Furthermore, rosette nanotubes (RNTs) are typically longer in reality (≈100 nm) than in the simulations, suggesting that when multiple drug molecules are confined within the inner channel, they may remain entrapped for longer durations before transitioning to the ends due to the extended length of the nanotube.

### Releasing mechanism in acidic environments

Regarding drug release, several studies have explored the release behavior of nanocarriers under acidic conditions through molecular simulations.^64–69^ A common approach involves protonating the nanocarrier to examine its structural behavior at low pH levels. A carbon nanotube conjugated with DNA fragments mainly composed of cytosine was employed in one study.^49^ The researchers studied the structural behavior of the nanocarrier in a protonated environment to better understand the release mechanism at low pH levels.^49^ They aimed to determine the behavior of the nanocarrier system when exposed to acidic environments. In this study, the G∧C motifs within each RNT were selectively protonated on the cytosine residues, following the approach described by the mentioned study.

**Fig. 14:**
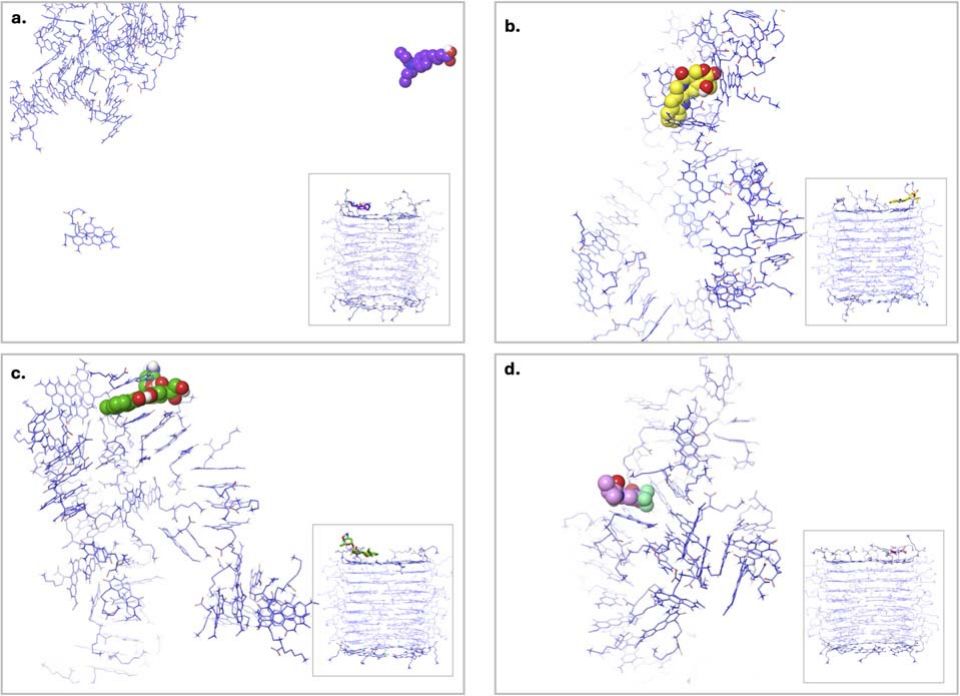
Clustered MD trajectories of the complex systems at pH 5.2, consisting of (a) CBL, (b) CPT, (c) DOX, and (d) FLU at the LysR site. The structures shown correspond to the clusters obtained from the last 10 ns of the simulation, with the first representative structure from each cluster displayed. The initial states used here were derived from the stable conformations of each complex under physiological conditions.

**Fig. 15:**
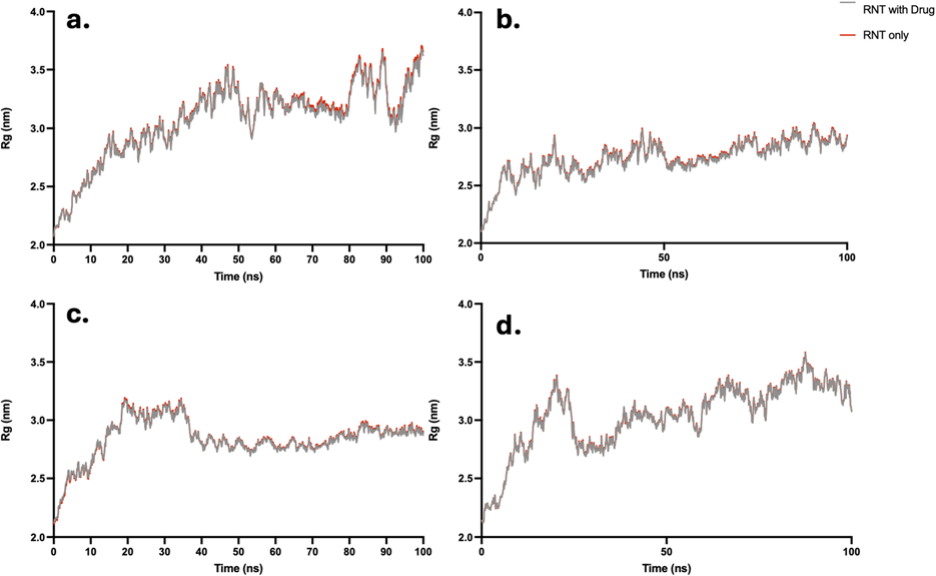
Radius of gyration analysis of the MD simulations of (a) CBL, (b) CPT, (c) DOX, and (d) FLU under acidic conditions.

Earlier, each drug exhibited stability when it remained at the LysR site of xK1. In this study, stable complex systems, where drugs remained at LysR, were protonated to elucidate the release mechanism. From the visual inspection of the MD trajectories (Fig. 14a–d and SI representative Video S13), xK1 began to disassemble within the first 10 ns, as evidenced by the increasing *R*_g_ (Fig. 15a–d) during this period. This behavior indicates the successful release of the drug from the RNT structure. Although some drug molecules appeared to interact with residual aggregates from the disrupted RNTs, the overall breakdown still reflects a release mechanism.

The inherent biocompatibility of the RNT motifs supports their nontoxic persistence within biological environments, as demonstrated in prior biomedical applications. For instance, RNTs were utilized for tissue engineering applications as a scaffold for neural regeneration and nerve repair.^31^ One study showed that RNT motifs are internalized *via* temperature-dependent endocytosis, confirming that endocytic pathways are primarily responsible for their cellular uptake.^30^ This suggests that, following RNT disassembly, target cells can efficiently absorb both the drug and the motif molecules through natural cellular mechanisms. Thus, an endocytosis mechanism is anticipated to occur within the target cancer cells.

## Conclusions

This study comprehensively investigated the structural and energetic stability of the xK1 type of RNT as a potential delivery vehicle for chemotherapeutic drugs under physiological conditions, with the purpose of reducing the adverse effects associated with these drugs. This study also investigated the structural response of RNT in acidic environments to elucidate the release mechanism. Molecular dynamics simulations, aided by robust energetic analysis, were conducted to assess the viability of xK1 as a drug carrier and to elucidate factors contributing to its stability in physiological environments. Results from *R*_g_, RMSD, and MM/3DRISM analyses revealed that the xK1 type of RNT exhibits both structural and energetic stability with CBL, CPT, DOX, and FLU as the drug molecules, under physiological conditions, with binding energies ranging from −4.66 to −24.10 kcal mol^−1^. Visual inspection further demonstrated that these drugs preferentially localize at the terminal regions of the xK1 structure, attributed to the prominent aromatic rings at both ends, which facilitate π–π stacking and π–cation interactions. On the other hand, to elucidate the mechanism of drug release within target cells, molecular dynamics simulations under acidic conditions were performed. Given that the tumor microenvironment is often characterized by acidic pH, drug release was investigated by protonating the stable RNT (pH = 5.1). Protonating the RNT at low pH levels confirmed the successful release of each drug following structural disassembly within the first 10 ns, supporting its potential as a responsive drug-delivery platform. These results offer a coherent basis for the experimental validation and future optimization of RNT-based nanocarriers.

## Author contributions

The manuscript was written through the contributions from all the authors. All authors have given approval to the final version of the manuscript. Hanah Nasifa M. Ali: conceptualization, data curation, formal analysis, funding acquisition, investigation, methodology, software, resources, validation, visualization, writing-original draft, and writing-review & editing. Arthur A. Gonzales III: conceptualization, data curation, formal analysis, funding acquisition, investigation, methodology, software, project administration, resources, software, supervision, validation, visualization, writing-original draft, and writing-review & editing.

## Conflicts of interest

There are no conflicts to declare.

## Data Availability

Data presented in this study are available in the article and the supplementary information (SI). Supplementary information is available. See DOI: https://doi.org/10.1039/d5ra04657b.
